# A Multidimensional approach to Severe Asthma Management: the need for Pulmonary Rehabilitation among candidates for Biologic Therapy

**DOI:** 10.3389/fmed.2026.1853986

**Published:** 2026-07-23

**Authors:** Michela Pozza, Gabriella Guarnieri, Andrea Vianello

**Affiliations:** Respiratory Pathophysiology Unit, Department of Cardiac-Thoracic-Vascular Sciences and Public Health University of Padova, Padova, Italy

**Keywords:** deconditioning, exertional dyspnea, frequent exacerbations, Th2-inflammation, treatable traits

## Abstract

**Background:**

Despite optimized pharmacological therapy, a significant subset of severe asthma patients remains poorly controlled. Lack of control is often driven by complex, non-inflammatory “treatable traits” that require a multidimensional management strategy beyond traditional airway targeting.

**Objective:**

To characterize clinical, functional, and inflammatory profile of severe asthma patients at the time of prescribing biologic therapy and identify potential candidates to ARM (Assessment-Rehabilitate-Maintenance) pulmonary rehabilitation (PR) program.

**Methods:**

This retrospective study analyzed 195 consecutive adults meeting GINA criteria for severe asthma admitted to our outpatient clinic between January 1, 2015, and December 31, 2025. ARM-program candidacy was predefined by the coexistence of: FEV1 < 80% predicted, mMRC ≥ 2, ACT < 20, and ≥2 annual exacerbations.

**Results:**

The median age of the cohort was 55 years and 60% of patients were female. A substantial disease burden was detected (median ACT: 16; annual exacerbations: 3). Females showed significantly higher maintenance oral corticosteroid (OCS) dependence (35.9% vs. 21.8%, *p* = 0.036) and increased risk of air trapping (RV/TLC: 42.0% vs. 38.5%, *p* = 0.035). 47 patients (24%) met ARM candidacy criteria, showing higher OCS use (42.6% vs. 26.4%; *p* = 0.035), and a four-fold higher prevalence of anxiety/depression (12.8% vs. 3.4%; *p* = 0.015) compared with non-candidates.

**Conclusions:**

At the time of biologic prescription, a significant proportion of severe asthma patients present a multidimensional burden, including dyspnea, frequent exacerbations, and psychological comorbidities and are potential candidates to ARM PR program. Prospective studies are needed to validate patient selection criteria and support whether pulmonary rehabilitation—may improve clinical and functional outcomes in target subgroup.

## 1 Background

Patients with severe asthma frequently remain poorly controlled despite maximal optimized pharmacological therapy, resulting in persistent symptoms, recurrent exacerbations, and repeated use of systemic corticosteroids (1). Although the introduction of biologic therapies has significantly improved disease management, severe asthma treatment continues to represent a substantial unmet clinical need.

To date, six biologic agents targeting key inflammatory pathways are available, including anti-IL-5/IL-5R (mepolizumab, reslizumab, benralizumab), anti-IL-4R (dupilumab), anti-TSLP (tezepelumab), and anti-IgE (omalizumab). These treatments have demonstrated robust efficacy in reducing exacerbation rates, hospitalizations, and corticosteroid exposure, while improving patients' quality of life (2). However, asthma remains a highly heterogeneous condition, in the context of which disease burden is not solely driven by airway inflammation, but also by extrapulmonary and behavioral components. This complexity has led to the recognition of “treatable traits” as key determinants of disease expression and therapeutic response (3).

As a result, contemporary asthma management is progressively shifting toward a multidimensional and personalized approach that integrates pharmacological therapies with non-pharmacological interventions. Among these, pulmonary rehabilitation (PR) has emerged as a promising strategy to address modifiable traits such as physical inactivity, deconditioning, dyspnea, and impaired functional status (1, 3, 4).

PR is a comprehensive intervention combining exercise training, education, and behavioral modification. Evidence suggests that PR may improve exercise capacity, symptom burden, and health-related quality of life, while reducing healthcare utilization and potentially limiting pharmacological escalation (5). Nevertheless, current evidence on its use in asthma remains heterogeneous in terms of program structure, delivery modalities, and patient selection, whilst clear referral criteria are still lacking. Recently, we proposed the ARM (Assessment-Rehabilitate-Maintenance) program, a structured, evidence-based PR pathway specifically tailored to address this gap, providing a structured, evidence-based rehabilitation program with defined eligibility criteria and core components (6). However, in real-world clinical practice, it remains unclear which patients with severe asthma are potential candidates to this intervention, particularly at the time of starting biologic therapy.

Aim: this study aims to describe the clinical and functional profile of patients with severe asthma prior to biologics starting and to identify baseline features associated with a higher likelihood of suitability for the ARM PR program. A better understanding of these characteristics may support a prompt and targeted implementation of rehabilitation strategies, contributing to a personalized and integrated management of severe asthma (5, 6).

## 2 Methods

This observational retrospective single-center, real-world cohort study included consecutive outpatients with severe asthma attending the Respiratory Pathophysiology outpatient clinic at the Azienda Ospedale–Università di Padova between January 1, 2015 and December 31, 2025. The analysis focused on data collected immediately before starting biologic therapy. The study had a descriptive and exploratory design. According to the eligibility criteria proposed for ARM pulmonary rehabilitation programme (6), patients were divided into two groups based on the presence or absence of all the following clinical and functional indicators: FEV_1_ < 80% predicted, mMRC ≥ 2, ACT score < 20, and at least two exacerbations in the previous year. This classification was used for descriptive subgroup analysis only and was not intended to represent a validated decision algorithm or to predict response to pulmonary rehabilitation. Requiring all four criteria identified a restrictive subgroup with airflow limitation, clinically relevant dyspnea, poor asthma control, and frequent exacerbations. The study was not designed to provide external validation of the ARM framework or to assess the clinical effectiveness of pulmonary rehabilitation. Because 6-min walk test data were not systematically available, the mMRC scale was used as a pragmatic indicator of perceived dyspnea-related limitation rather than as a direct measure of objective exercise capacity or deconditioning. Ethical approval was released by the local Ethics Committee (Cesc 5724/AO/23, URC AOP2937). Study procedures followed an internal hospital protocol approved by the Regional Health Authority, in accordance with the Declaration of Helsinki. After obtaining written informed consent, the patient's data were collected.

### 2.1 Study population

Patients were considered eligible for inclusion if they met the following criteria: age ≥ 18 years; confirmed diagnosis of severe asthma according to the Global Initiative for Asthma (GINA) criteria (uncontrolled asthma despite optimized treatment corresponding to GINA treatment steps 4–5; therapy adherence, correct inhaler technique and presence of modifiable factors were assessed).

Patients with asthma-COPD overlap, bronchiectasis, or other respiratory diseases were included.

The following exclusion criteria were applied: patients already enrolled in structured pulmonary rehabilitation programs; comorbidities with contraindications to pulmonary rehabilitation, such as severe neurological conditions or cardiocirculatory instability; patients who had already initiated biologic therapy.

### 2.2 Data collection

Baseline anthropometric, clinical, and functional data were collected at the visit performed to assess eligibility for biologic therapy.

The following variables were considered:

- demographic data: age, sex, body mass index (BMI)
- lifestyle factors: smoking status
- disease characteristics: disease duration, pharmacological treatment, and history of exacerbations (moderate exacerbation: an episode thet requires change in treatment/use of OCS; severe exacerbation: an episode requiring emergency visits or hospitalization).
- comorbidities: numbers and treatable traits according to Agusti et al. (7). In particular: a) pulmonary traits (upper airway inflammation:rhinitis, rhinosinusitis; nasal polyposis, allergies, bronchiectasis, other respiratory diseases: including COPD, OSAS, interstitial lung diseases, emphysema.), b) extra-pulmonary traits (T2 inflammation: blood eosinophil count >200 cells/μl and/or FeNO >25 ppb, GERD, cardiovascular diseases, obesity, osteoporosis/osteopenia, anxiety/depression:documented physician diagnosis), c) behavioral traits (smoking status).
- pulmonary function parameters: FEV_1_, FEV_1_/FVC ratio, and RV/TLC ratio
- inflammatory biomarkers: eosinophilic counts, fractional exhaled nitric oxide (FeNO), IgE

Measures related to functional status and symptom burden were also collected, including:

- Asthma Control Test (ACT)
- Modified Medical Research Council dyspnea scale (mMRC)

### 2.3 Statistical analysis

ARM-programme candidate group and non- ARM-programme candidate group were compared; moreover, an age-based subgroup analyses was conducted, using the median age of the study population as the cut-off value. Baseline data were expressed as median and quartiles (Q1–Q3) for continuous variables, or as counts and percentages for categorical variables. The normality of data distribution was assessed using the Shapiro–Wilk test. Initial stratification of the study population was performed according to gender and then was based on ARM criteria. Comparisons between groups were performed using the Mann-Whitney U test for continuous variables and the Chi-square test or Fisher's exact test, as appropriate, for categorical variables. Statistical significance was set at *p* < 0.05. A Bonferroni correction was applied to adjust for multiple comparisons, with the significance threshold set at *p* < 0.05/n. Univariable and multivariable binomial logistic regression models were used to explore the discriminatory performance of the predefined ARM criteria, individually and in combination. AUC, sensitivity, and specificity were assessed at a probability threshold of 0.5, and these analyses were considered exploratory rather than externally validating the ARM framework. In addition, an exploratory multivariable binomial logistic regression model was fitted to identify factors associated with ARM-programme candidacy beyond the variables directly included in the ARM definition. This model included age, sex, comorbidity burden, OCS use, GERD, anxiety/depression, osteoporosis/osteopenia, and BMI as covariates. Missing data were reported for all variables included in the analysis. No imputation was performed, and analyses were conducted using available data only. Complete data were available for all variables used to define ARM-programme candidacy. Statistical analysis was performed using Jamovi [(version 2.7.14) Jamovi project, Sydney, Australia].

## 3 Results

The study population included 195 patients, 117 females (60%) and 78 males (40%), with an average median age of 55 years, slightly overweight, predominantly non-smokers, with a long history of asthma and an average of 4 comorbidities. 145 patients (74.4%) were classified as having uncontrolled asthma according to ACT criteria. The mMRC-dyspnea score was 2 (Q1-Q3: 1–2). Most clinical characteristics showed no significant sex-related differences (Table 1). However, female patients showed a significantly higher prevalence of daily maintenance oral corticosteroid (OCS) use compared with males (35.9% vs. 21.8%- *p* = 0.036), a greater number of exacerbations in the last year (*p* = 0.002), and increased air trapping (*p* = 0.035). Analysis of comorbidities revealed a number of sex-related differences. Chronic rhinosinusitis with nasal polyps (CRSwNP) and obstructive sleep apnea were significantly more prevalent in male patients [42 (53.85%) vs. 44 (37.61%); *p* = 0.025 and 6 (7.69%) vs. 2 (1.71%); *p* = 0.039, respectively], whereas osteoporosis/osteopenia was significantly more common in female patients [32 (27.35%) vs. 8 (10.26%); *p* = 0.004].

**Table 1:** Baseline characteristics.

Characteristic	Data avaiable (*n*)	Male (*n* = 78)	Female (*n* = 117)	Tot. (*n* = 195)	*p* value
Age-year	195	56 (46.3–64)	55 (46–64)	55 (46–64)	0.954
Body mass index	192	25.6 (23.5–28)	25.2 (21.8–29.2)	25.3 (22.3–28.7)	0.289
Smoking status-no (%)	195				0.037
Never smokers		63/78 (80.8)	105/117 (89.7)	168/195 (86.2)	0.075
Former smokers		15/78 (19.2)	10/117 (8.5)	25/195 (12.8)	**0.029**
Current smokers		0 (0.0)	2/117 (1.7)	2/195 (1.0)	0.518
Disease duration (years)	130	21 (7.5–33.0)	20 (10.0–29.5)	20.5 (9.25–30.0)	0.808
Comorbidities-no	195	4 (3–5)	4 (2–5)	4 (3–5)	0.319
Background medication-no. (%)	195				
Triple therapy (ICS+LABA+LAMA)		59/78 (75.6)	99/117 (84.6)	158/195 (81.0)	0.117
Maintenance Oral corticosteroids (OCS)		17/78 (21.8)	42/117 (35.9)	59/195 (30.3)	**0.036**
Leukotriene receptor antagonists (LTRAs)		29/78 (37.2)	47/117 (40.2)	76/195 (39)	0.675
Allergy medications		32/78 (41.0)	46/117 (39.3)	78/195 (40)	0.811
Respiratory support		2/78 (2.6)	4/117 (3.4)	6/195 (3.1)	0.735
Biomarkers of type 2 inflammation					
Blood eosinophil count- cells^*^μl	141	540 (210–850)	460 (220–793)	520 (220–810)	0.544
FeNO-ppb	125	38.3(19.9–59.5)	33.3 (17.9–59.6)	35 (18.1–60.0)	0.720
**Lung function**					
Pre-bronchodilator FEV_1_-liters	195	2.71 (1.69–3.29)	1.86 (1.38–2.30)	2.1 (1.5–2.82)	**<0.001**
Pre-bronchodilator FEV_1_-% predicted	195	78.5 (56.3–94.0)	78.0 (58.0–93.0)	78.0 (58.0–93.5)	0.982
Pre-bronchodilator FEV_1_/FVC-%	168	68.0 (61.5–76.8)	73.0 (63.3–80.2)	71.0 (62.0–79.0)	0.051
RV/TLC -%	151	38.5 (32.0–47.0)	42 (36.5–49.6)	41 (33.9–49.0)	**0.035**
Asthma control test	195	16.5 (12.3–20.0)	16.0 (12.0–19.0)	16.0 (12.0–20.0)	0.607
Controlled asthma (ACT ≥ 20)		21/78 (26.9)	29/117 (24.8)	50/195 (25.6)	0.738
Uncontrolled asthma (ACT < 20)		57/78 (73.1)	88/117 (75.2)	145/195 (74.4)	0.738
mMRC	195	2 (1–2)	2 (1–2)	2 (1–2)	0.501
No. of moderate or severe asthma exacerbations in previous yr	195	2 (1–3)	3 (2–4)	3 (2–4)	**0.002**

Data are presented as median (Q1–Q3) for continuous variables and as counts (percentages) for categorical variables. Percentages are calculated within each group. Variables related to background medication are not mutually exclusive. Comparisons between groups were performed using the Mann–Whitney U test for continuous variables and the Chi-square test or Fisher's exact test, as appropriate. Bold values indicate statistically significant results (*p* < 0.05.)

**Figure 1:**
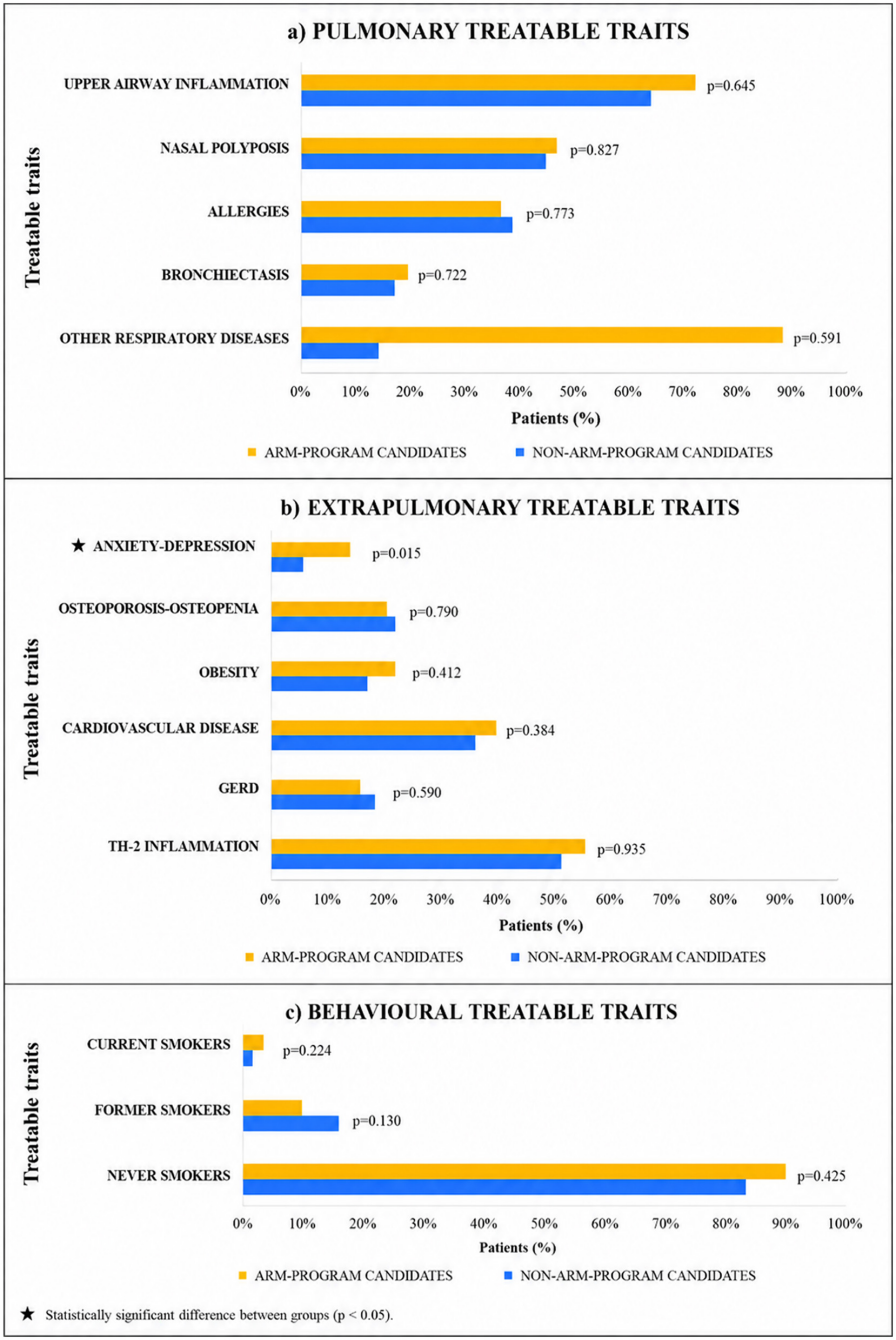
Treatable trait profiles in ARM and non-ARM program candidates. Proportion (%) of treatable traits in ARM-program candidates and non-ARM-program candidates determined by multidimensional assessment for: **(a)** pulmonary traits, **(b)** extra-pulmonary traits, **(c)** behavioral. Values represent the percentage of patients with at least one condition within each domain. Significant difference in trait prevalence between ARM-candidates and non-ARM candidates*.

Patients were stratified into two groups: ARM-program candidates (*n* = 47) and non-ARM-program candidates (*n* = 148; Table 2). The first group showed higher OCS use (42.6% vs. 26.4%, *p* = 0.035), more compromised functional status (with a difference in median value of Pre-bronchodilator FEV_1_ about 0.75L), a double number of exacerbations and a difference of 4 points in median ACT score compared with the other group. No significant differences were found in T2 inflammation biomarkers between groups. The comorbidity profile was largely comparable between groups, except for anxiety and depression, which were significantly more prevalent in the ARM-candidates group (12.77% vs. 3.38%, *p* = 0.015; Figure 1).

**Table 2:** Characteristics of ARM-program candidates and non-ARM program candidates.

Characteristic	ARM-program candidates (*n* = 47)	Non-ARM-program candidates (*n* = 148)	*p* value
Age-year	52 (43.5–62.5)	56.5/148 (48.0–64.0)	0.117
Male sex-no. (%)	17/47 (36.2)	61/148 (41.2)	0.538
Body mass index	26.4 (23.3–28.9)	25.3 (22.2–28.6)	0.266
Disease duration (years)	20 (10–26)	21 (9–30)	0.864
Comorbidities-no	3 (3–5)	4 (2.75–5)	0.799
**Background medication-no. (%)**			
Triple therapy (ICS+LABA+LAMA)	41/47 (87.2)	117/148 (79.1)	0.213
Maintenance Oral corticosteroids (OCS)	20/47 (42.6)	39/148 (26.4)	**0.035**
Leukotriene receptor antagonists (LTRAs)	20/47 (42.6)	56/148 (37.8)	0.564
Allergy medications	21/47 (44.7)	57/148 (38.5)	0.452
Respiratory support	3/47 (6.4)	3/148 (2.0)	0.132
**Biomarkers of type 2 inflammation**			
Blood eosinophil count- μl	635 (335–955)	480 (200–790)	0.081
FeNO-ppb	44.7 (26.8–66.0)	33.0(17.7–58.0)	0.186
**Lung function**			
Pre-bronchodilator FEV_1_-liters	1.53 (1.27–2.06)	2.28 (1.67–3.06)	**<0.001**
Pre-bronchodilator FEV_1_-% predicted	58.0 (49.0–69.0)	85.0 (67.8–98.3)	**<0.001**
Pre-bronchodilator FEV_1_/FVC-%	66.5 (59.3–72.8)	73.0 (64–80)	**0.008**
RV/TLC -%	44.2 (37.0–51.0)	40.2 (33.0–47.4)	0.087
Asthma Control Test	14 (11–17)	18 (13–21)	**<0.001**
mMRC	2 (2–3)	1 (1–2)	**<0.001**
No. of moderate or severe asthma exacerbations in previous yr	4 (3–5)	2 (1–3)	**<0.001**

Data are presented as median (Q1–Q3) for continuous variables and as counts (percentages) for categorical variables. Percentages are calculated within each group. Variables related to background medication are not mutually exclusive. Comparisons between groups were performed using the Mann–Whitney *U* test for continuous variables and the Chi-square test or Fisher's exact test, as appropriate. Bold values indicate statistically significant results (*p* < 0.05.

Overall, 47 patients met all four criteria, while 110 and 167 patients fulfilled at least three or at least two criteria, respectively. Exploratory analyses assessing the discriminatory performance of the individual ARM criteria and their combined use are reported in Supplementary Table S1. Overall, the combined model including FEV_1_% predicted, mMRC score, ACT score, and exacerbation burden showed higher discriminatory performance and improved specificity compared with the individual criteria.

In the exploratory multivariable logistic regression model, ARM-programme candidacy remained associated with higher BMI and anxiety/depression after adjustment for selected clinical covariates. The model was statistically significant overall, although with modest explanatory value (χ^2^ = 16.2, *p* = 0.039; McFadden *R*^2^ = 0.077).

Age-stratified analyses (< 55 vs. ≥55 years) were performed in both groups to evaluate the impact of age in comorbidities and physical ability. Among ARM-program candidates, older patients (≥55 years) showed greater air trapping (increased RV/TLC, 50% vs. 39% *p* = 0.013) and a higher proportion of former smokers (3 vs. 0; *p* = 0.017; Supplementary Table S2). They also showed a higher prevalence of osteoporosis/osteopenia [7 (41.18%) vs. 2 (6.67%); *p* = 0.004] and hypertension [6 (12.8%) vs. 3 (10.0%); *p* = 0.034), whereas allergic disease was more frequent in younger patients [14 (46.47%) vs. 3 (17.65%); *p* = 0.047]. In the non-ARM group, older patients had higher BMI (25.9 vs. 23.6; *p* = 0.025), greater comorbidity burden (4 vs. 3; *p* = 0.029), and a smoking profile characterized by a higher number of former smokers [17 (19.8%) vs. 5 (8.1%); *p* = 0.048]. They also exhibited higher blood eosinophil counts (540 vs. 340; *p* = 0.012) and worse lung function (lower FEV/FVC-−68.8% vs. 78.1%, *p* < 0.001, and higher RV/TLC-−45% vs. 33.1%; *p* < 0.001). Rhinitis and allergic disease were more common in younger patients, whereas nasal polyposis [45 (52.33%) vs. 20 (32.26%); *p* = 0.015] and osteoporosis/osteopenia [23 (26.74%) vs. 8 (12.9%); *p* = 0.041] were more prevalent in older individuals.

## 4 Discussion

Investigating patients with severe asthma prior to biologic initiation, this retrospective cohort study identified a predominantly female population with a high disease burden. Patients showed poor asthma control with frequent exacerbations, clinically relevant dyspnea, and persistent symptoms despite optimized pharmacological therapy in nearly three-quarters of cases. Female patients exhibited greater disease severity, with higher rates of maintenance OCS use, more frequent exacerbations, and increased air trapping. These findings are consistent with previous reports describing poorer control and higher exacerbation burden in women with severe asthma (8).

In the absence of specific criteria for referral to pulmonary rehabilitation in severe asthma, we prioritized patients with great clinical impairment, including high symptom burden, great functional limitation, and poor disease control, in accordance with previous studies (6). Our analyses indicate that the identification of ARM-program candidates is highly dependent on the restrictiveness of the eligibility definition. While the primary four-criterion definition selected a smaller subgroup with greater multidimensional impairment, less restrictive definitions identified a substantially larger proportion of patients. These findings support the use of the current definition as a conservative, hypothesis-generating approach, but also highlight the need for prospective validation of ARM referral criteria.

Because 6-min walk test (6MWT) data were not systematically available, the mMRC scale was used as a surrogate of perceived functional limitation, supported by evidence from COPD populations showing a strong association with 6-min walk distance (6MWD) (9). Our results support the potential role of PR within a treatable trait framework. Indeed, candidates to PR showed poor asthma control, severe dyspnea, frequent exacerbations, OCS dependence, and a higher prevalence of anxiety and depression, indicating persistent unmet needs despite optimized pharmacological treatment. Moreover, older ARM candidates showed more severe air trapping, a higher number of comorbidities, and were more commonly former smokers, while younger patients had more allergic disease. This profile highlights a clear opportunity for PR as a personalized, multidisciplinary intervention targeting symptom burden, functional limitation, deconditioning, psychological distress, physical inactivity, and self-management (7, 10).

The clinical profile of ARM-program candidates is consistent with previous studies. Jarosch et al. (11) reported that patients referred to pulmonary rehabilitation were typically younger, more symptomatic, and had poorer disease control, greater exertional dyspnea, and more depressive symptoms. In our cohort, ARM-program candidates also demonstrated a significantly higher rate of anxiety and depression, emphasizing the relevance of psychological distress as an extrapulmonary treatable trait in severe asthma. In fact, emotional distress has been associated with reduced physical activity and sedentary behavior, even in clinically stable patients (12). Although evidence on the impact of PR on psychological outcomes in asthma remains heterogeneous, our program incorporated psychological support and education addressing emotional wellbeing, consistent with previous approaches (6). A multidisciplinary strategy may also address nutritional aspects, as ARM-program candidates tended to have higher BMI, although this difference was not statistically significant (13).

**Figure 2:**
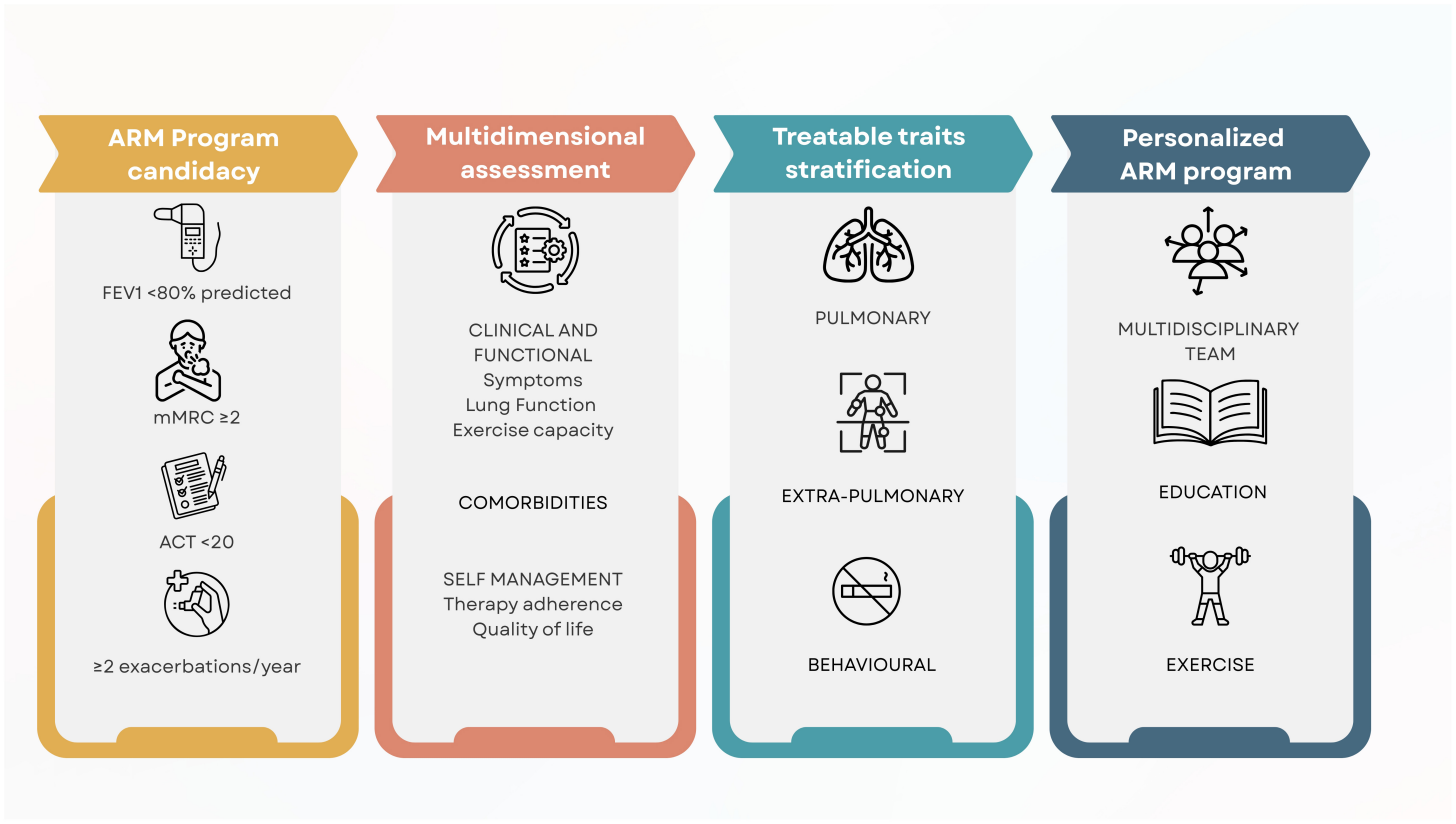
Conceptual framework of the ARM programme for identifying candidates for pulmonary rehabilitation in severe asthma.

Our findings do not indicate that PR should delay or replace biologic therapy. Rather, they identify a subgroup of patients with severe asthma who, at the time of biologic therapy assessment, present multidimensional impairment and treatable traits potentially relevant to future evaluation of PR as an adjunctive intervention. This perspective is consistent with the GINA framework, which recommends optimization of asthma management before biologic therapy, including non-pharmacological interventions alongside assessment of adherence, inhaler technique, and comorbidities (1). Prospective interventional studies are needed to determine whether PR has any impact on subsequent treatment escalation, including biologic therapy use (Figure 2) (5, 6).

This strategy may be particularly relevant in patients who are not optimal candidates for biologics or whose disease burden is not fully explained by T2 inflammation, as biologic therapies may not adequately target the broader multidimensional aspects of severe asthma (2).

From an economic perspective, pulmonary rehabilitation is among the most cost-effective interventions in chronic respiratory diseases, particularly in COPD (14). Although asthma-specific cost-effectiveness data are limited, PR may reduce both direct and indirect costs, including healthcare utilization and work absenteeism (15–18).

This study has several limitations. ARM candidacy was defined using clinical and functional criteria rather than standardized guideline definitions and therefore requires prospective validation. The single-center design may limit generalizability of our findings, as referral pathways, biologic availability, PR infrastructure, and patient characteristics may differ across healthcare systems. External validation in multicentre cohorts is therefore needed before the proposed ARM-candidacy definition can be applied more broadly. In addition, the use of surrogate measures and the absence of direct assessments of exercise capacity, such as the 6MWT, may have influenced ARM classification, as mMRC alone cannot fully capture deconditioning, exercise intolerance, quality of life, treatment adherence, and socioeconomic factors may have caused underestimation of relevant treatable traits. The lack of data on work absenteeism also limited evaluation of indirect costs. Finally, the relatively small sample size may have reduced statistical power, and findings of the study may exclusively apply to patients assessed at the time of biologic therapy initiation.

## 5 Conclusion

At the time of biologic treatment initiation, patients with severe asthma exhibited a high baseline clinical burden despite optimized therapy, with poor disease control, frequent exacerbations, and clinically relevant dyspnea. Female patients showed greater disease severity, including higher use of oral corticosteroids and increased exacerbation rates. A distinct subgroup was identified with multidimensional impairment extending beyond inflammatory phenotype, characterized by more severe symptoms, functional limitation, and a higher prevalence of psychological comorbidities. These findings highlight important unmet needs that cannot be fully addressed by pharmacological treatment. In this context, pulmonary rehabilitation may represent a multidisciplinary intervention with the potential to address deconditioning, dyspnea, and extrapulmonary treatable traits. Its early integration in severe asthma care plan, is consistent with guideline-recommended optimization of care. Prospective studies are needed to validate patient selection criteria and support whether pulmonary rehabilitation—may improve clinical and functional outcomes in target subgroup.

## Data Availability

The raw data supporting the conclusions of this article will be made available by the authors, without undue reservation.
