# Supplementary material for: A Multidimensional approach to Severe Asthma Management: the need for Pulmonary Rehabilitation among candidates for Biologic Therapy
Source: Front Med (Lausanne). 2026 Jul 23;13:1853986. doi: 10.3389/fmed.2026.1853986 (PMC13442376; doi:10.3389/fmed.2026.1853986)
Supplement: Supplementary file 1 [file Table_1.docx]

| **Model** | **Predictor(s) included** | **AUC** | **Sensitivity** | **Specificity** |
| --- | --- | --- | --- | --- |
| Univariable model | ACT score | 0.692 | 0.959 | 0.043 |
| Univariable model | Exacerbation burden | 0.749 | 0.959 | 0.106 |
| Univariable model | mMRC score | 0.845 | 0.892 | 0.468 |
| Univariable model | FEV1 % predicted | 0.816 | 0.919 | 0.255 |
| Multivariable combined model | ACT score, mMRC score, FEV1 % predicted, exacerbation burden | 0.930 | 0.953 | 0.660 |

**Supplementary Table S1 - Exploratory discriminatory performance of individual ARM criteria and combined model for ARM-programme candidacy**

AUC, sensitivity, and specificity were derived from univariable and multivariable binomial logistic regression models assessing ARM-programme candidacy. Sensitivity and specificity were calculated at the default probability threshold of 0.5. The combined model included all four predefined ARM criteria.

**Curva ROC**

Receiver operating characteristic curve of the combined multivariable model for ARM-programme candidacy. The model included ACT score, mMRC score, FEV1 % predicted, and exacerbation burden.
