# Supplementary material for: A Multidimensional approach to Severe Asthma Management: the need for Pulmonary Rehabilitation among candidates for Biologic Therapy
Source: Front Med (Lausanne). 2026 Jul 23;13:1853986. doi: 10.3389/fmed.2026.1853986 (PMC13442376; doi:10.3389/fmed.2026.1853986)
Supplement: Supplementary file 2 [file Table_2.docx]

| Characteristic | ARM PROGRAM CANDIDATES  <55 (N=30) | ARM PROGRAM CANDIDATES  ≥55 (N=17) | p value | NON-ARM PROGRAM CANDIDATES  <55 (N=62) | NON-ARM PROGRAM CANDIDATES  ≥55 (N=86) | p value |
| --- | --- | --- | --- | --- | --- | --- |
| Upper airway and allergic diseases |  |  |  |  |  |  |
| Rhinitis | 10 (33.33) | 3 (17.65) | 0.248 | 33 (53.23) | 17 (19.77) | **<0.001** |
| Rhinosinusitis | 10 (33.33) | 7 (41.18) | 0.591 | 13 (20.97) | 24 (27.91) | 0.336 |
| Nasal polyposis | 17 (56.67) | 9 (52.94) | 0.805 | 20 (32.26) | 45 (52.33) | **0.015** |
| Allergic disease | 14 (46.67) | 3 (17.65) | **0.047** | 39 (62.90) | 18 (20.93) | **<0.001** |
| Allergic Bronchopulmonary Aspergillosis (ABPA) | 2 (6.67) | 0 (0.00) | 0.277 | 1 (1.61) | 4 (4.65) | 0.313 |
| Respiratory comorbidities |  |  |  |  |  |  |
| Bronchiectasis | 5 (16.67) | 4 (23.53) | 0.566 | 9 (14.52) | 16 (18.60) | 0.512 |
| Chronic obstructive pulmonary disease (COPD) | 0 (0.00) | 0 (0.00) | - | 0 (0.00) | 2 (2.33) | 0.510* |
| Emphysema | 0 (0.00) | 0 (0.00) | - | 1 (1.61) | 2 (2.33) | 1.000* |
| Alpha-1 antitrypsin deficiency | 0 (0.00) | 1 (2.1) | 0.362* | 1 (1.61) | 2 (2.33) | 1.000* |
| Obstructive sleep apnoea (OSA) | 1 (3.33) | 0 (0.00) | 1.000* | 2 (3.23) | 5 (5.81) | 0.464 |
| Relevant comorbidities |  |  |  |  |  |  |
| Obesity | 6 (20.0) | 4 (23.53) | 0.822 | 8 (12.90) | 16 (18.60) | 0.359 |
| Eosinophilic Granulomatosis with Polyangiitis (EGPA) | 3 (10.0) | 5 (29.41) | 0.089 | 8 (12.90) | 16 (18.60) | 0.353 |
| Gastroesophageal reflux disease (GERD) | 4 (13.33) | 3 (17.65) | 0.690 | 8 (12.90) | 18 (20.93) | 0.205 |
| Anxiety and depression | 4 (13.33) | 2 (11.76) | **0.877** | 2 (3.23) | 3 (3.49) | 0.930 |
| Osteoporosis-osteopenia | 2 (6.67) | 7 (41.18) | **0.004** | 8 (12.90) | 23 (26.74) | **0.041** |
| Hypertension | 3 (10.0) | 6 (12.8) | **0.034** | 10 (16.13) | 23 (26.74) | 0.126 |
| Diabetes | 1 (3.33) | 0 (0.00) | 1.000* | 2 (3.23) | 6 (6.98) | 0.319 |
| Dyslipidaemia | 4 (13.33) | 0 (0.00) | 0.281 | 4 (6.45) | 14 (16.28) | 0.071 |
| Other comorbidities |  |  |  |  |  |  |
| Respiratory diseases | 2 (6.67) | 0 (0.00) | 0.528* | 3 (4.84) | 1 (1.16) | 0.309* |
| ENT diseases | 0 (0.00) | 0 (0.00) | - | 3 (4.84) | 3 (3.49) | 0.681 |
| Cardiovascular diseases | 5 (16.67) | 7 (14.9) | 0.064 | 11 (17.74) | 18 (20.93) | 0.630 |
| Gastrointestinal diseases | 2 (6.67) | 4 (23.53) | 0.096 | 8 (12.90) | 21 (24.42) | 0.082 |
| Neurological diseases | 2 (6.67) | 2 (11.76) | 0.613* | 5 (8.06) | 7 (8.14) | 0.987 |
| Renal diseases | 0 (0.00) | 1 (5.88) | 0.362* | 1 (1.61) | 3 (3.49) | 0.640* |
| Musculoskeletal diseases | 3 (10.0) | 2 (11.76) | 0.850 | 2 (3.23) | 7 (8.14) | 0.217 |
| Eye diseases | 1 (3.33) | 1 (5.88) | 1.000* | 2 (3.23) | 6 (6.98) | 0.319 |
| Urogenital diseases | 2 (6.67) | 4 (23.53) | 0.096 | 5 (8.06) | 15 (17.44) | 0.329 |
| Skin diseases | 1 (3.33) | 1 (5.88) | 1.000* | 3 (4.84) | 5 (5.81) | 0.487 |
| Endocrine diseases | 2 (6.67) | 1 (5.88) | 1.000* | 6 (9.68) | 14 (16.28) | 0.246 |
| Rheumatologic diseases | 0 (0.00) | 0 (0.00) | - | 1 (1.61) | 3 (3.49) | 0.640* |
| Immunodeficiency | 1 (3.33) | 0 (0.00) | 1.000* | 2 (3.23) | 1 (1.16) | 0.572 |
| Cancer | 2 (6.67) | 2 (11.76) | 0.613* | 2 (3.23) | 8 (9.30) | 0.146 |

**Supplementary Table S2: Age-stratified analyses (<55 vs ≥55 years) between ARM-program candidates non-ARM candidates**

Data are presented as median (Q1–Q3) for continuous variables and as counts (percentages) for categorical variables. Percentages are calculated within each group. Comparisons between age-stratified groups were performed using the Mann–Whitney U test for continuous variables and the Chi-square test or Fisher’s exact test, as appropriate. P-values (*) are unadjusted; statistical significance was determined after Bonferroni correction.
